# Pathway-based drug repositioning using causal inference

**DOI:** 10.1186/1471-2105-14-S16-S3

**Published:** 2013-10-22

**Authors:** Jiao Li, Zhiyong Lu

**Affiliations:** 1 Institute of Medical Information, Chinese Academy of Medical Sciences, Beijing, China; 2 National Center for Biotechnology Information (NCBI), National Institutes of Health, Bethesda, USA

## Abstract

**Background:**

Recent *in vivo *studies showed new hopes of drug repositioning through causality inference from drugs to disease. Inspired by their success, here we present an *in silico *method for building a causal network (CauseNet) between drugs and diseases, in an attempt to systematically identify new therapeutic uses of existing drugs.

**Methods:**

Unlike the traditional 'one drug-one target-one disease' causal model, we simultaneously consider all possible causal chains connecting drugs to diseases via target- and gene-involved pathways based on rich information in several expert-curated knowledge-bases. With statistical learning, our method estimates transition likelihood of each causal chain in the network based on known drug-disease treatment associations (e.g. bexarotene treats skin cancer).

**Results:**

To demonstrate its validity, our method showed high performance (AUC = 0.859) in cross validation. Moreover, our top scored prediction results are highly enriched in literature and clinical trials. As a showcase of its utility, we show several drugs for potential re-use in Crohn's Disease.

**Conclusions:**

We successfully developed a computational method for discovering new uses of existing drugs based on casual inference in a layered drug-target-pathway-gene- disease network. The results showed that our proposed method enables hypothesis generation from public accessible biological data for drug repositioning.

## Background

Despite the fast growth in drug research and development(R&D) such as chemical genomics technologies [1,2] and chemical libraries [3,4], the pharmaceutical R&D output--new drugs brought to market--has significantly declined in recent decades. As reported in the most recent analysis, the number of new drugs approved per billion US dollars spent has halved approximately every 9 years since 1950 [5]. Discovering new uses for existing drugs, also known as drug repositioning, provides one possible solution to such a problem. The fact that existing drugs have already passed through development stages such as target validation and ADMET (absorption, distribution, metabolism, excretion and toxicity) characteristics analysis should greatly help reduce time and risk when attempting to identify their new indications [6].

The traditional one drug-one target-one disease drug discovery model has been argued to more likely result in poor efficacy or unanticipated side effects by not taking into account the complexity of underlying mechanism [7,8]. Due to such limitations, network-based computational approaches were proposed recently, providing a new framework for identifying drug-repositioning opportunities. Keiser *et al*. predicted new targets for known drugs using drug chemical structures and their canonical biological targets, and the resulting novel drug-target network further connected drugs to new indications [9]. Li *et al*. measured drug pairwise similarity by combining similarity of drug chemical structures, similarity of target profiles, and interaction between target proteins [10]. Iorio *et al*. constructed a drug-drug similarity network using transcriptional responses (*i.e*., gene expression profiles) following drug treatment [11]. Recent studies [12-14] compared the drug vs. disease gene expression profiles for identifying novel treatment relationships between drugs and diseases. Other kinds of network-based approaches for drug repositioning included literature mining [15] and shared pathway analysis [16].

Different from the aforementioned computational approaches, several recent studies demonstrated the feasibility of drug repositioning through manual analysis of causal associations in drug-involved pathways [17-20]. For example, Cramer *et al*. found that FDA approved anticancer drug bexarotene could be potentially used for Alzheimer's Disease (AD) treatment [19] based on molecular pathway examination and analysis. More specifically, they found bexarotene activates nuclear receptors PPAR (peroxisome proliferator-activated receptor) and LXR (liver × receptor) in coordination with RXR (retinoid × receptor), thus up-regulating the expression of the ApoE (apoliporrotein E) gene. This process facilitates the clearance of Aβ (β-amyloid) from the brain, resulting in the alleviation of AD. In this example, the chain of causality between one drug and one disease was examined and inferred by domain experts who took advantage of the following knowledge in bexarotene-related pathways: (1) drug-target (*e.g*., bexarotene is an RXR agonist); (2) target involved pathway (e.g., LXR:RXR activation pathway); (3) transcriptional responses in a given pathway (e.g., increased ApoE gene expression in the LXR:RXR activation pathway); (4) genetic mechanism of disease (e.g., ApoE is associated with AD).

Motivated by the success of manual pathway analysis for drug repositioning, we developed a new computational method for building a network of causal chains between drugs and diseases, allowing for computational drug repositioning. By taking advantage of the increasing amount of expert-curated biological knowledge in the public domain (e.g. pathway information in Pathway Commons [21]), we built a multi-layer causal network (CauseNet) consisting of chains from drug to target, target to pathway, pathway to downstream gene, and gene to disease. Furthermore, we used a statistical method to learn the transition likelihood of each causal chain in the network based on those known drug-disease treatment relationships. In the prediction stage, we identified novel drug re-uses using maximum likelihood estimation. Unlike the traditional causal chain models that relied on human examination of one drug target, pathway and gene at a time, our computational model allows us to investigate all possible causal links when connecting drugs to diseases at once. To our best knowledge, this is also the first attempt of using network-based causal inference in computational drug repositioning.

## Methods

**Figure 1:**
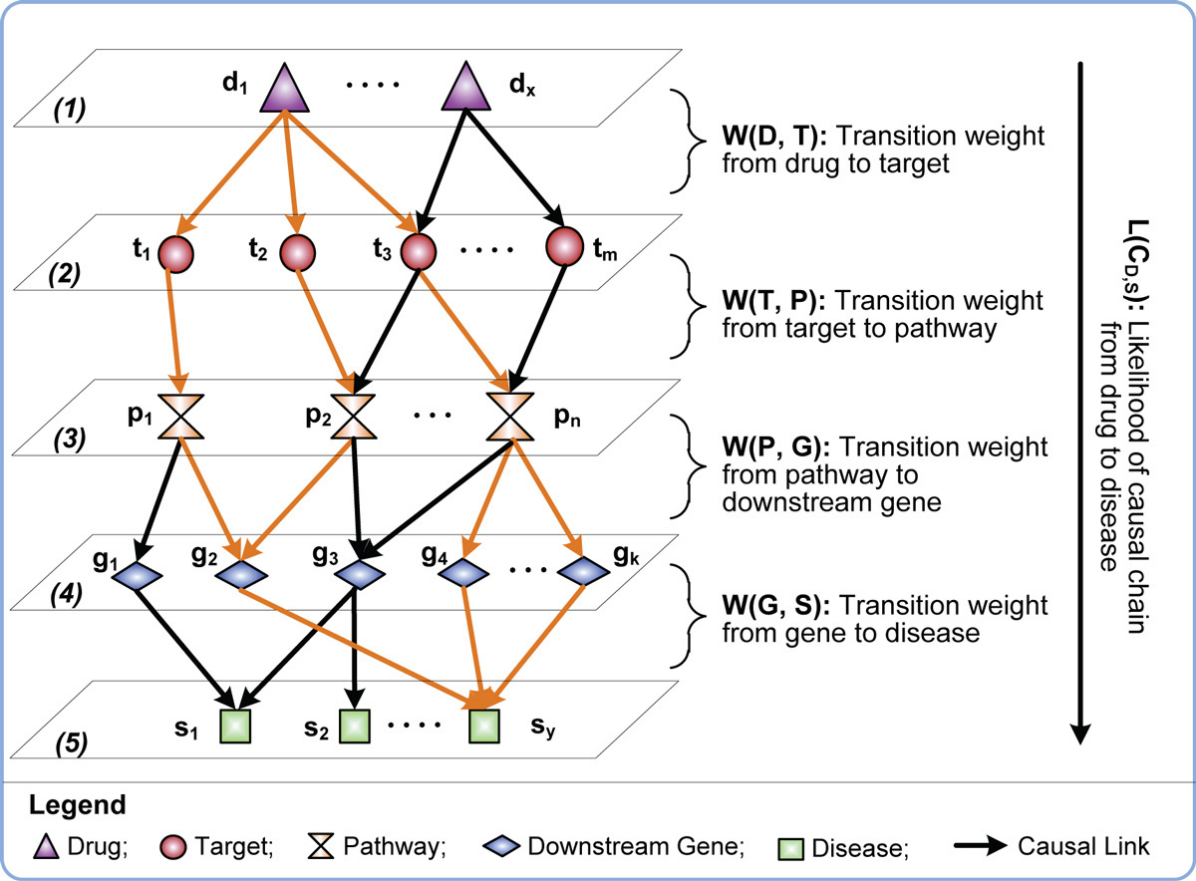
**A network-based view of causality between drugs and diseases**.

In Figure 1, we show a model of our proposed CauseNet which puts causal chains from drugs to diseases in a layered network. The nodes of CauseNet are organized in five layers: drug *D {d_1_, ...d_x_}*, target *T {t_1_, ...t_m_}*, pathway *P {p_1_, ...p_n_}*, downstream genes *G {g_1_, ..., g_k_}*, and disease *S {s_1_, ...s_y_}*. Accordingly, from top to bottom the causal links between two layers represent (1) drug *d *acts on target *t*; (2) target *t *participants in pathway *p*; (3) pathway *p *affects the expression of downstream gene *g*; and (4) gene *g *is associated with disease *s*. To construct such a network, we integrated data from heterogeneous resources which contain expert-curated knowledge of relationships between drugs, molecules and diseases. Furthermore, we learn the transition weight for each causal link in the CauseNet to distinguish the likelihood of transitions between nodes based on the known treatment relationships between drugs and diseases (details in Section computing transition weights). For instance, if drug *d_1 _*is known to treat disease *s_y_*, then the transition weights of the gold-colored links in Figure 1 should be promoted accordingly.

### Constructing CauseNet

For constructing CauseNet, we extracted approved drugs and their targets from DrugBank [22], target-involved-pathways from Pathway Commons [21] and KEGG [23], downstream genes from Pathway Commons, and diseases and their associated genes from Comparative Toxicogenomics Database (CTD) [24]. Also from CTD, we assembled pairs of known drug-disease treatment relationships. Note that each pathway can mention information on a series of biological events such as biochemical reactions, physical interactions, transcriptional responses, and phosphorylation and enzyme catalysis. In this study, we focused on transcriptional responses (*i.e*., up/down regulated expression of downstream genes) in a pathway.

### Computing transition weights

We represent the constructed CauseNet as a directed graph *G(V, E)*. The node set, *V(G)={D, T, P, G, S}*, consists of five types of objects (*i.e.*, drug *D*, target *T*, pathway *P*, downstream gene *G *and disease *S*). The edge set is denoted as *E(G) ⊆{D × T, T×P, P×G, G×S}*. A complete causal chain, *c = <d, t, p, g, s>*, represents a 4-step path from drug *d *(*d ∈ D*) to disease s (*s∈S*)with a set of individual chains *E(c) *= {*(d, t), (t, p), (p, g), (g, s)*}⊂*E(G)*. All possible causal chains from drugs to diseases become the complete chain set *C*. We further use a subset of (treatment-enriched) chains C* (*i.e*., C*⊂*C*) to represent the links between drug-disease pairs of known treatment relationships. For example, as shown in Figure 1, drug *d_1 _*is linked to diseases *s_2 _*and *s_y _*through two separatechains *c_1_=<d_1_, t_2_, p_2_, g_3_, s_2 _*> and *c_2_=<d_1_, t_2_, p_2_, g_2_, s_y_*>, where *c_1_, c_2_∈ C *and c_2_∈ C*(d_1 _is known to treat *s_y _*but not *s_2_)*.

The graphs of the respective complete and enriched chain sets *C *and C* are denoted as *G(C) = G(V(C), E(C)) *and G(C*) = G(V(C*), E(C*)), where V(C*) ⊂ V(C) and E(C*) ⊂ E(C). Given above, we can learn the transition weight *w(v_i_, v_j_) *to represent the transition likelihood from node to towards treatment relationships (∃*(v_i_, v_j_)∈E(C)*):

(1) $$w\left(v_{i},v_{j}\right)=\left\{\begin{array}{c} 1+\frac{p\left(v_{i}\to v_{j}|\;G\left(C^{*}\right)\right)}{p\left(v_{i}\to v_{j}|\;G\left(C\right)\right)}\;\;\;if\;\left(v_{i},v_{j}\right)\in E\left(C^{*}\right)\\ 1\;\;\;\;otherwise \end{array}\right)$$

Where *p*(*v_i _*→ *v_j_*|*G*(C*)) and *p*(*v_i _*→ *v_j_*|*G*(*C*)) are the transition probabilities from node *v_i _*to node *v_j _*in G(C*) and *G(C)*, respectively. Let each chain graph *G*(•) be a Markov model. Thus the transition probability *p*(*v_i_*→*v_j_*|*G*(•)) is computed using maximum likelihood estimation:

(2) $$p\left(v_{i}\to v_{j}\;|\;G\left(∙\right)\right)=\frac{Nv_{i},v_{j}}{Nv_{i},∙}$$

*Nv_i_*, *v_j _*is the number of times that a transition *v_i _*→ *v_j _*is observed in a chain set, and *Nv_i_*,• is the total number of transitions originated from *v_i _*in the chain set.

### Predicting novel treatment relationships between drugs and diseases

For each causal chain *c = <d, t, p, g, s>*in the global chain set (*c∈C*), we can estimate its likelihood *L(c) *based on the pre-computed transition weights in equation (1).

(3) L(c)=log(w(d,t)⋅w(t,p)⋅w(p,g)⋅w(g,s))

Our prediction of a new indication of drug *d_x _*for disease *s_y _*is based on the final score *S*(*d_x_*, *s_y_*) between drug *d_x _*and disease *s_y_*, which is the maximal likelihood of all possible chains from *d_x _*to *s_y_*:

(4) $$S\left(d_{x},s_{y}\right)=\text{max}\left(L\left(c_{x,y}\right)\right),\;\;\;\;\;\;c_{x,y}\in \left\{<d_{x},t_{∙},p_{∙},g_{∙},s_{y}>\right\}$$

*c_x,y _*is a causal chain from drug *d_x _*to disease *s_y _*among all possible chains $C_{x,y}=\left\{<d_{x},t_{\cdot },p_{{}_{\cdot }},g_{{}_{\cdot }},s_{y}>\right\}$. Note that alternatively, *S(d_x_,s_y_) *can also be measured simply by the number of successful chains from *d_x _*to *s_y_*: *|C_x,y_|*. As shown below, we used such a method as a baseline for comparing our weighted method.

## Results

### Complete and treatment-enriched chain sets

Based on the CauseNet (see Section constructing CauseNet), we constructed a complete causal chain set *C *including 2,711,440 possible 4-step chains from 979 drugs, to 538 targets, to 207 pathways, to 1,122 downstream genes, to 1,650 diseases, corresponding to 389,945 possible drug-disease associations. A total of 6,268 such associations between 665 drugs and 583 diseases were labelled as known (i.e. found in CTD), resulting in a total of 135,936 chains to the treatment-enriched chain subset C*.

**Figure 2:**
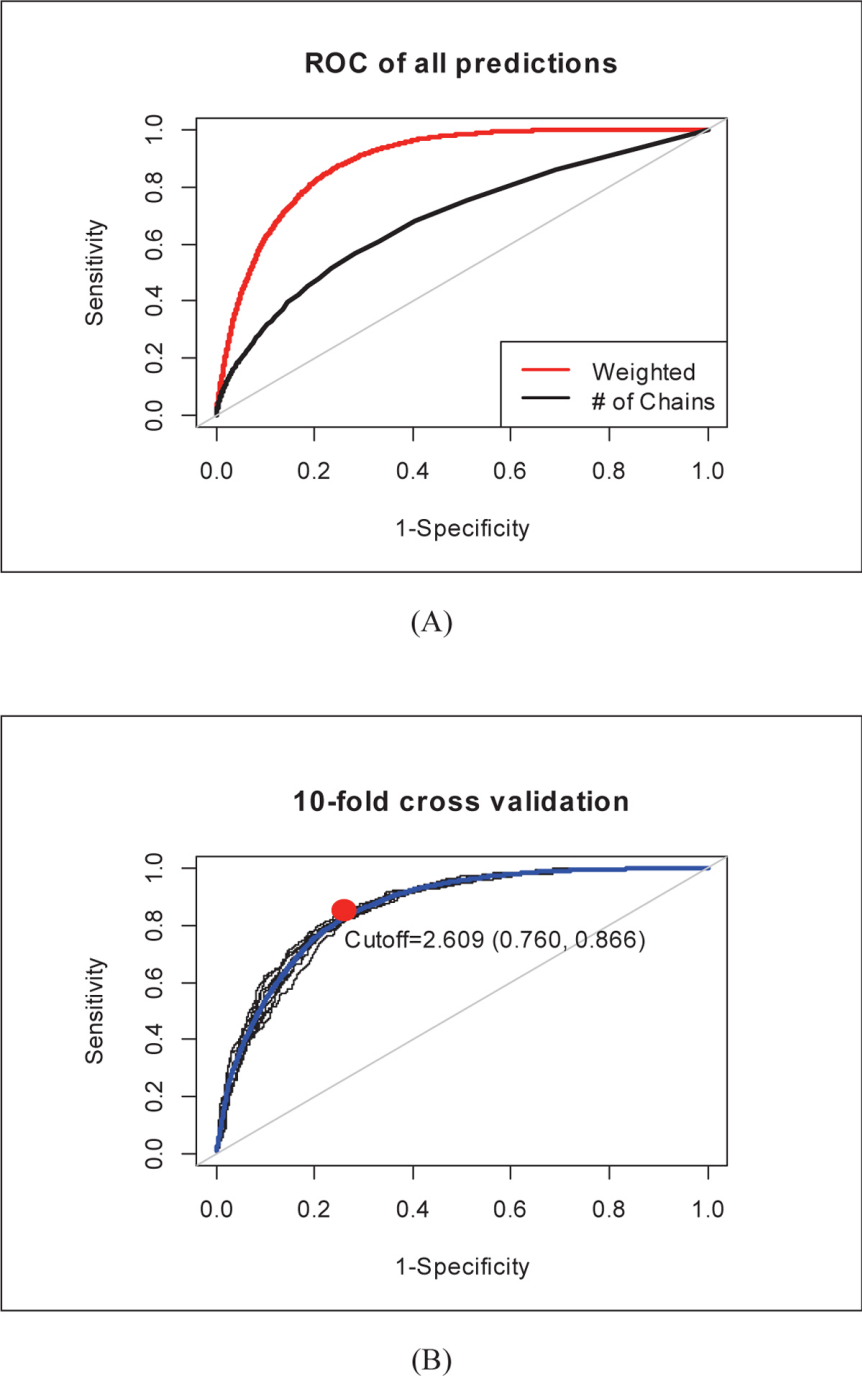
**ROC curves of our methods in predicting therapeutic effects**.

Table 1 shows detailed statistics of the complete vs. enriched chain sets and their corresponding graph elements. For each edge in *G(C)*, we calculated its transition weight based on equation 1 (see Section computing transition weights). Furthermore, we computed scores for each of the 389,945 possible drug-disease associations based on the maximal likelihood estimation of causal chains (equation 4) and ranked them accordingly. When treating the known 6,268 associations as the only positive instances, we calculated true positive rate (sensitivity) and false positive rate (1-specificity) of our results at different cut-off ranking scores. As plotted as a ROC curve in Figure 2(A), we obtained a high AUC score of 0.889, which suggests that the 6,289 known (positive) associations were indeed ranked high among all 389,945 pairs. Also in Figure 2(A), we show that our weighted inference method significantly outperformed the baseline method in AUC scores, which shows the value of computing weights for transition between nodes in our CauseNet.

**Table 1:** Descriptive statistics of global and treatment enriched chain sets

		Complete chain set *C*	Enriched chain subset C*
**# of chains**		2,711,440	135,936

**Node set**	**# of drugs *|D|***	979	655
	
	**# of targets *|T|***	538	397
	
	**# of pathways *|P|***	207	199
	
	**# of genes *|G|***	1,122	838
	
	**# of diseases *|S|***	1,650	583

**Edge set**	***|D×T|***	2,953	2,074
	***|T×P|***	2,922	2,004
	
	***|P×G|***	2,772	2,179
	
	***|G×S|***	6,496	3,954

**# of drug-disease associations**		389,945	6,268

### Cross validation of therapeutic effect prediction

To further evaluate the validity of our method, we conducted a 10-fold cross validation by withholding 10% of the known treatment relationships in each fold and removing their connected chains accordingly. Figure 2 (B) shows the results of all ten ROC curves, with the average AUC score of 0.859 ± 0.006 with (CI = 0.95) (highlighted in blue). The best tradeoff between sensitivity (0.866) and specificity (0.760) is shown in red, which corresponds to 2.609 in our prediction score. After filtering known ones, 92,057 associations between 964 drugs and 1050 diseases have scores higher than 2.609. Additional File 1 lists the 92,057 predicted associations and all possible causal chains connecting the drug-disease associations via target-and gene-involved pathways.

**Figure 3:**
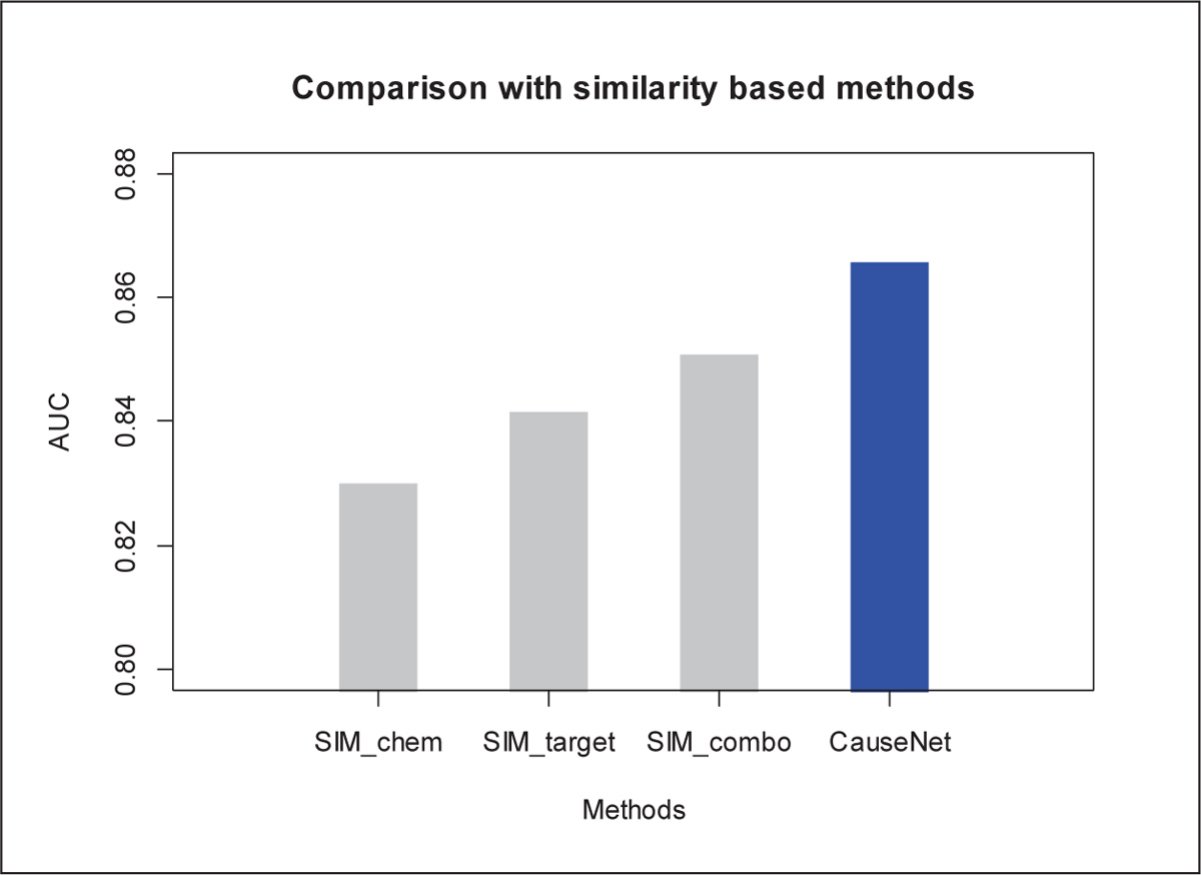
**Comparison with similarity-based methods in predicting therapeutic effects**.

We compared our method with the similarity-based methods [9,10] which assume that similar drugs are used for similar diseases' treatments. Drug pairwise similarity was measured by chemical 2D structure similarity (SIM_chem), drug target similarity (SIM_target), and linear combination of these two (SIM_combo) respectively. We applied the similarity-based methods to 602 small molecule drugs (with 2D chemical structure) in our CauseNet dataset. As can be seen in Figure 3, our method achieved a higher AUC score (0.866) than using chemical similarity (0.829), target similarity (0.841) or their combination (0.851).

### Novel predictions in clinical trials and literature

We further evaluated our predictions by searching evidence in clinical trials and literature. About 1/3 were found in PubMed [25] (requiring three or more occurrences) and a relatively small percentage of our predictions (3,202) were found in ClinicalTrials.gov [26]. There are several main reasons for more evidence in the literature than in clinical trials: First, some predicted therapeutic uses are still in pre-clinical development and hence have not reached clinical trial stage. For example, we predicted anakinra to treat colorectal neoplasm with a high confidence score of 5.996. According to literature evidence [27], anakinra--a drug approved for treating rheumatoid arthritis--was recently found to be able to contribute to growth-inhibition of small tumors in mice with colon carcinoma. Second, clinical trials are not always registered in ClinicalTrails.gov. In our results, some highly scored predictions were found for novel uses of nadroparin--a drug outside of the U.S. market. Some trials have been launched for investigating these new uses in countries outside the U.S., with their studies reported in literature, but not in ClinicalTrials.gov.

**Figure 4:**
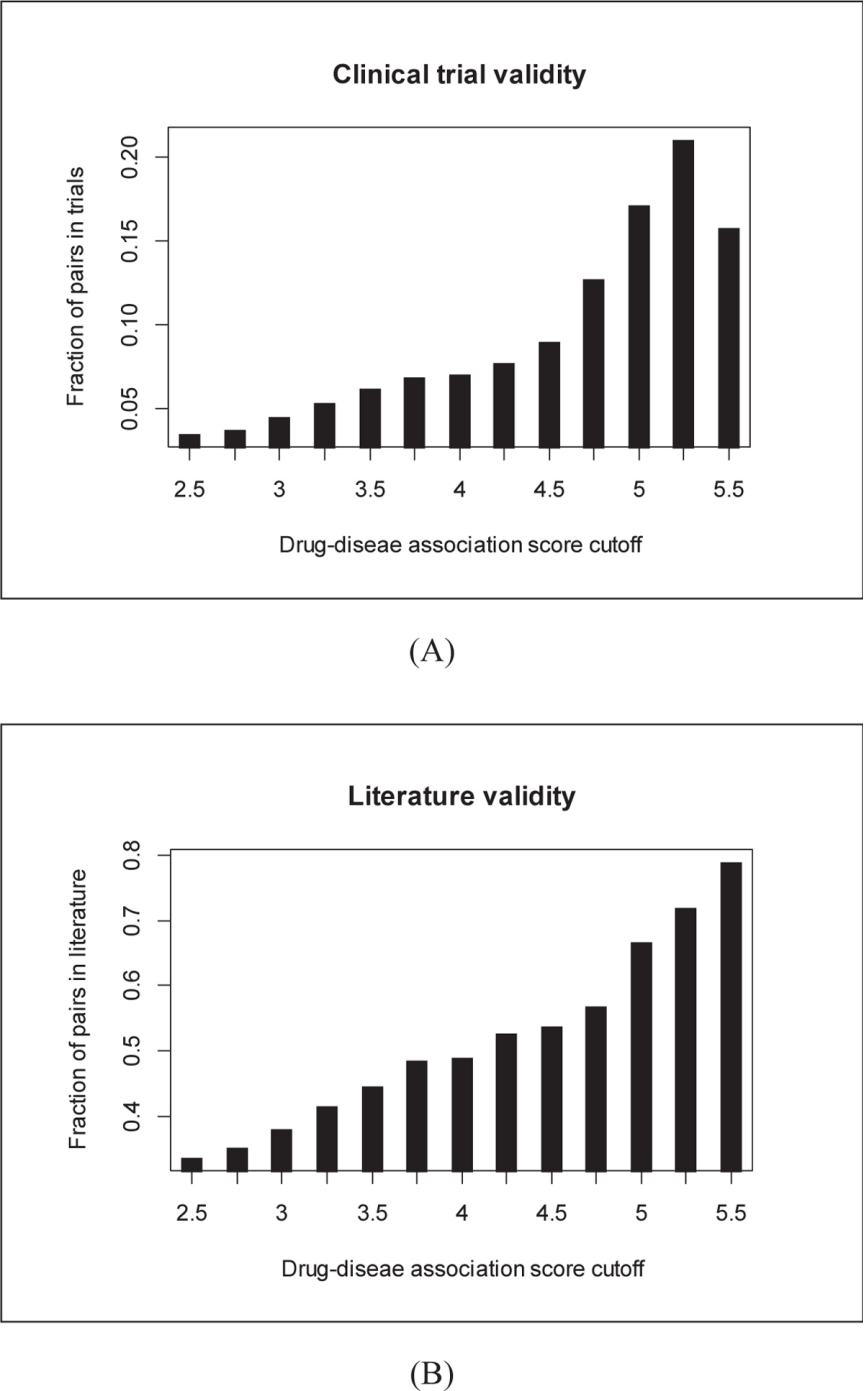
**Clinical trial and literature validity of novel drug-disease association predictions**.

To demonstrate the discriminative power of our prediction scores, we show in Figure 4 that in general the higher the prediction score and more likely the predicted association can be validated in ongoing clinical trial investigations and scientific publications. Hence, we believe such a score can greatly help others to use our prediction results for further investigations.

### Investigations of drug repositioning opportunities for Crohn's Disease

Drug repositioning for poorly treated diseases is a promising strategy in drug discovery today because of the highly unmet need there [5]. In this study, we further explored drug repositioning opportunities for Crohn's disease (CD), a chronic inflammatory condition of the gastrointestinal tract, for which there is no known cure and most treatment options aim to relieve its symptoms such as rectal bleeding and diarrheal [28]. Every year, 10,000 ~ 47,000 residents of North America are diagnosed with CD, and as many as 630,000 currently suffer from CD [29]. Epidemiology studies showed incidence of CD is highly influenced by geographic region and family history. Recently, genetic efforts have been made to explain these epidemiologic observations and to understand the underlying pathogenesis from the view of human genomics [30,31]. As a result, multiple CD susceptibility genes have been found such as IL23R, IL6, IL10, NLRP3, FN1, NCF4 and FPR2. These findings could lead to identifying novel therapeutic options for CD.

**Figure 5:**
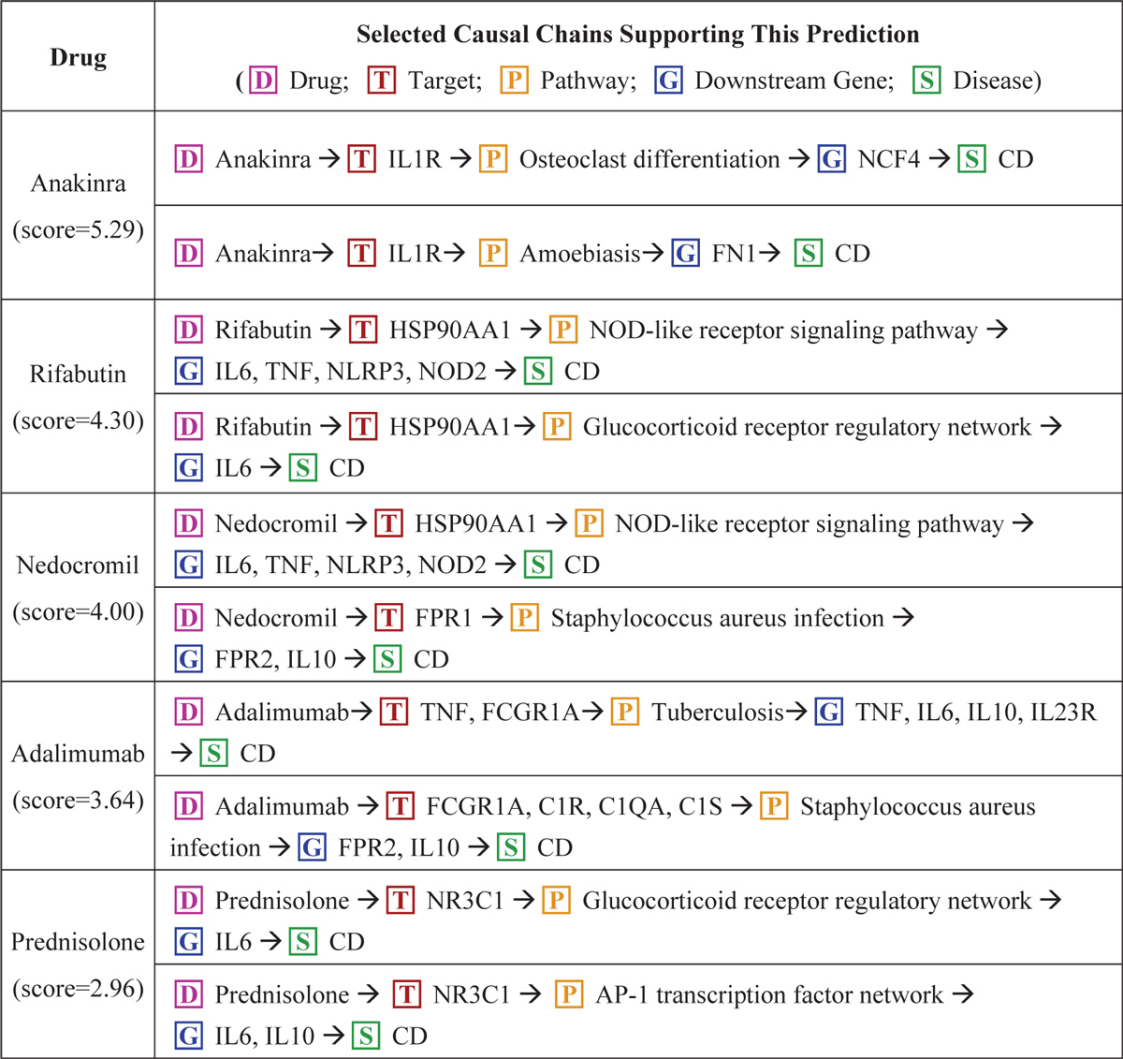
**Potential drugs for Crohn's Disease (CD) treatment**.

Figure 5 shows five selected CD drugs predicted by our method for CD and their exemplar causal chains found in our CauseNet. For example, anakinra, an approved rheumatoid arthritis drug, shows a high potential for CD treatment with a score of 5.26 in our method. Further analysis shows that anakinra works by binding receptor IL1R, which may influence multiple pathways like osteoclast differentiation pathway and amoebiasis pathway, affecting CD genes NCF4 and FN1 respectively. Another highly scored drug is nedocromil (score = 4.00), a drug approved for treating allergic conjunctivitis and asthma. Our method shows its potential therapeutic use in CD through acting on multiple targets HSP90AA1 and FPR1, affecting multiple pathways NOD-like receptor signaling pathway and staphylococcus aureus infection pathway, and further affecting multiple CD mechanism genes IL6, TNF, NLRP3, NOD2, FPR2 and IL10. This comprehensive evidence would greatly help experts generate hypotheses on the therapeutic values of these CD drug candidates which are worth further experimenting. We find that two drugs shown in Figure 5, adalimumab and prednisolone, have also been previously studied for CD [32,33].

## Discussion

In this study, we propose a new computational drug repositioning approach by using causal chains in drug-disease networks (see Figure 1). Our method has the following important characteristics:

First, it provides a broad and semantic view of molecular causality between drugs and diseases. Unlike the traditional 'one drug-one target-one disease' model, we put all causality relationships between drugs and disease in a network view with five distinct layers. In the CauseNet construction, we integrated different types of data and semantic relationships between them from widely recognized and expert-curated resources. For example, when integrating pathway data, we focused on specific direction (downstream) and specific semantics (transcriptional response) relationships in an interested pathway by taking advantage of recent progress in pathway curation and standardization [21,34]. The resulting CauseNet laid down a key foundation for further drug-disease relationship prediction.

Second, not only does our method find novel drug-disease treatment associations, but also scores and ranks each prediction accordingly. As shown in the cross-validation experiment, our method is able to rank true associations generally at the top positions. Moreover, those highly scored drug-disease prediction results are found significantly enriched in clinical trials and biomedical literature. Hence, we believe that our weighted inference method is able to prioritize prediction results for further exploring drug repositioning opportunities.

Third, instead of being a black box, our method provides detailed and comprehensive molecular evidence supporting each prediction. As shown in the case study with Crohn's disease, the accompanying pathway evidence can support further human investigation. More importantly, such comprehensive pathway information could reveal unknown linkages between drugs and disease and help hypothesis generation on novel drug re-uses.

Lastly, our prediction results cover a wide range of diseases and drugs. For drugs, our repositioning results consist of both small molecule drugs (*e.g*., rifabutin) and big molecules (*e.g.*, adalimumab), thus lifting the limitations of those methods that rely on 2D chemical structures or gene expression profiles of small molecules [9-14]. In addition, our method can identify drugs for a disease with no current treatments, making it different from similarity-based methods where predictions are always based on known uses of other drugs.

Like other knowledge-based methods, our approach relies on existing knowledge of drug-target, target-pathway, pathway-downstream gene, gene-disease, and drug-disease relationships. Despite increasing efforts in data curation and standardization, at present such information is still incomplete, thus limiting the prediction power of our method. For example, we extracted 1,239 target-involved pathways, but merely 209 of which contain transcriptional response relationships. Combining gene expression with pathway analysis to predict downstream genes is a hopeful strategy to help break the bottleneck [35]. We plan to investigate this issue in future work.

## Conclusions

In this study, we successfully developed a computational drug repositioning method using pathway-based causal inference. Unlike the traditional 'one drug-one target-one disease' causal model, we systematically considered all possible causal chains connecting drugs to diseases via target- and gene-involved pathways. More specifically, we built a multi-layer causal network (CauseNet) consisting of chains from drugs to disease by integrating heterogeneous expert-curated biological resources in public domain. The transition likelihood of each causal edge in the CauseNet was estimated by learning known drug-disease treatment relationships. Furthermore, we predicated novel drug indications using maximum likelihood estimation of causal chains between drugs and diseases. In cross-validation experiments, our method achieved AUC score of 0.859 ± 0.006 with best tradeoff sensitivity = 0.866 and specificity = 0.760. When compared with a control group of drug uses, our drug repositioning results were found to be significantly enriched in both the biomedical literature and clinical trials. Additionally, in the Crohn's Disease case study, we demonstrated our method would provide more comprehensive evidence showing how drugs connect to diseases via pathways. We believe our method would greatly help experts generate hypotheses in drug discovery.

## Competing interests

The authors declare that they have no competing interests.

## Authors' contributions

JL and ZL conceived the whole study, participated in its design, analyzed the results and wrote the manuscript. JL collected the data, implemented the methods and performed the experiments. All authors read and approved the final manuscript.

## Supplementary Material

Additional file 1 **Predicted drug-disease associations**. lists the 92,057 predicted associations and all possible causal chains connecting the drug-disease associations via target-and gene-involved pathways Click here for file

## Declarations

Publication of this article was funded by the Intramural Research Program of the National Institutes of Health.

This article has been published as part of *BMC Bioinformatics *Volume 14 Supplement 16, 2013: Twelfth International Conference on Bioinformatics (InCoB2013): Bioinformatics. The full contents of the supplement are available online at http://www.biomedcentral.com/bmcbioinformatics/supplements/14/S16.

## References

[B1] Kim DH Sim T Chemical kinomics: a powerful strategy for target deconvolution BMB Rep 2010 14 11 711 719 10.5483/BMBRep.2010.43.11.711 21110913

[B2] Roemer T Davies J Giaever G Nislow C Bugs, drugs and chemical genomics Nat Chem Biol 2012 14 1 46 56 10.1038/nnano.2012.218 22173359

[B3] Wang Y Xiao J Suzek TO Zhang J Wang J Bryant SH PubChem: a public information system for analyzing bioactivities of small molecules Nucleic Acids Res 2009 14 Web Server W623 633 10.1093/nar/gkp456 19498078 PMC2703903

[B4] Gaulton A Bellis LJ Bento AP Chambers J Davies M Hersey A Light Y McGlinchey S Michalovich D Al-Lazikani B ChEMBL: a large-scale bioactivity database for drug discovery Nucleic Acids Res 2012 14 Database D1100 1107 21948594 10.1093/nar/gkr777 PMC3245175

[B5] Scannell JW Blanckley A Boldon H Warrington B Diagnosing the decline in pharmaceutical R&D efficiency Nat Rev Drug Discov 2012 14 3 191 200 10.1038/nrd3681 22378269

[B6] Ashburn TT Thor KB Drug repositioning: identifying and developing new uses for existing drugs Nat Rev Drug Discov 2004 14 8 673 683 10.1038/nrd1468 15286734

[B7] Dudley JT Schadt E Sirota M Butte AJ Ashley E Drug discovery in a multidimensional world: systems, patterns, and networks J Cardiovasc Transl Res 2010 14 5 438 447 10.1007/s12265-010-9214-6 20677029

[B8] Schadt EE Friend SH Shaywitz DA A network view of disease and compound screening Nat Rev Drug Discov 2009 14 4 286 295 10.1038/nrd2826 19337271

[B9] Keiser MJ Setola V Irwin JJ Laggner C Abbas AI Hufeisen SJ Jensen NH Kuijer MB Matos RC Tran TB Predicting new molecular targets for known drugs Nature 2009 14 7270 175 181 10.1038/nature08506 19881490 PMC2784146

[B10] Li J Lu Z A New Method for Computational Drug Repositioning Using Drug Pairwise Similarity Proceedings of The IEEE International Conference on Bioinformatics and Biomedicine (BIBM) 2012 Philadelphia, USA 10.1109/BIBM.2012.6392722 PMC4175719 25264495

[B11] Iorio F Bosotti R Scacheri E Belcastro V Mithbaokar P Ferriero R Murino L Tagliaferri R Brunetti-Pierri N Isacchi A Discovery of drug mode of action and drug repositioning from transcriptional responses Proc Natl Acad Sci USA 2010 14 33 14621 14626 10.1073/pnas.1000138107 20679242 PMC2930479

[B12] Hu G Agarwal P Human disease-drug network based on genomic expression profiles PLoS One 2009 14 8 e6536 10.1371/journal.pone.0006536 19657382 PMC2715883

[B13] Sirota M Dudley JT Kim J Chiang AP Morgan AA Sweet-Cordero A Sage J Butte AJ Discovery and preclinical validation of drug indications using compendia of public gene expression data Sci Transl Med 2011 14 96 96ra77 10.1126/scitranslmed.3001318 21849665 PMC3502016

[B14] Shigemizu D Hu Z Hung JH Huang CL Wang Y DeLisi C Using functional signatures to identify repositioned drugs for breast, myelogenous leukemia and prostate cancer PLoS Comput Biol 2012 14 2 e1002347 10.1371/journal.pcbi.1002347 22346740 PMC3276504

[B15] Li J Zhu X Chen JY Building disease-specific drug-protein connectivity maps from molecular interaction networks and PubMed abstracts PLoS Comput Biol 2009 14 7 e1000450 10.1371/journal.pcbi.1000450 19649302 PMC2709445

[B16] Li Y Agarwal P A pathway-based view of human diseases and disease relationships PLoS One 2009 14 2 e4346 10.1371/journal.pone.0004346 19194489 PMC2631151

[B17] Strittmatter WJ Medicine. Old drug, new hope for Alzheimer's disease Science 2012 14 6075 1447 1448 10.1126/science.1220725 22442467

[B18] Sivachenko A Kalinin A Yuryev A Pathway analysis for design of promiscuous drugs and selective drug mixtures Curr Drug Discov Technol 2006 14 4 269 277 10.2174/157016306780368117 17430103

[B19] Cramer PE Cirrito JR Wesson DW Lee CY Karlo JC Zinn AE Casali BT Restivo JL Goebel WD James MJ ApoE-directed therapeutics rapidly clear beta-amyloid and reverse deficits in AD mouse models Science 2012 14 6075 1503 1506 10.1126/science.1217697 22323736 PMC3651582

[B20] Kotelnikova E Yuryev A Mazo I Daraselia N Computational approaches for drug repositioning and combination therapy design J Bioinform Comput Biol 2010 14 3 593 606 10.1142/S0219720010004732 20556864

[B21] Cerami EG Gross BE Demir E Rodchenkov I Babur O Anwar N Schultz N Bader GD Sander C Pathway Commons, a web resource for biological pathway data Nucleic Acids Res 2011 14 Database D685 690 10.1093/nar/gkq1039 21071392 PMC3013659

[B22] Wishart DS Knox C Guo AC Cheng D Shrivastava S Tzur D Gautam B Hassanali M DrugBank: a knowledgebase for drugs, drug actions and drug targets Nucleic Acids Res 2008 14 Database D901 906 18048412 10.1093/nar/gkm958 PMC2238889

[B23] Kanehisa M Goto S Furumichi M Tanabe M Hirakawa M KEGG for representation and analysis of molecular networks involving diseases and drugs Nucleic Acids Res 2010 14 Database D355 360 10.1093/nar/gkp896 19880382 PMC2808910

[B24] Davis AP Murphy CG Saraceni-Richards CA Rosenstein MC Wiegers TC Mattingly CJ Comparative Toxicogenomics Database: a knowledgebase and discovery tool for chemical-gene-disease networks Nucleic Acids Res 2009 14 Database D786 792 10.1093/nar/gkn580 18782832 PMC2686584

[B25] PubMed^®^ http://www.ncbi.nlm.nih.gov/pubmed/

[B26] ClinicalTrials.gov http://clinicaltrials.gov/

[B27] Harnack U Johnen H Pecher G IL-1 receptor antagonist anakinra enhances tumour growth inhibition in mice receiving peptide vaccination and beta-(1-3),(1-6)-D-glucan Anticancer Res 2010 14 10 3959 3965 21036709

[B28] Crohn's Disease http://digestive.niddk.nih.gov/ddiseases/pubs/crohns/

[B29] Loftus EV Jr Clinical epidemiology of inflammatory bowel disease: Incidence, prevalence, and environmental influences Gastroenterology 2004 14 6 1504 1517 10.1053/j.gastro.2004.01.063 15168363

[B30] Rioux JD Xavier RJ Taylor KD Silverberg MS Goyette P Huett A Green T Kuballa P Barmada MM Datta LW Genome-wide association study identifies new susceptibility loci for Crohn disease and implicates autophagy in disease pathogenesis Nat Genet 2007 14 5 596 604 10.1038/ng2032 17435756 PMC2757939

[B31] Kenny EE Pe'er I Karban A Ozelius L Mitchell AA Ng SM Erazo M Ostrer H Abraham C Abreu MT A genome-wide scan of Ashkenazi Jewish Crohn's disease suggests novel susceptibility loci PLoS Genet 2012 14 3 e1002559 10.1371/journal.pgen.1002559 22412388 PMC3297573

[B32] Dudley JT Sirota M Shenoy M Pai RK Roedder S Chiang AP Morgan AA Sarwal MM Pasricha PJ Butte AJ Computational repositioning of the anticonvulsant topiramate for inflammatory bowel disease Sci Transl Med 2011 14 96 96ra76 10.1126/scitranslmed.3002648 21849664 PMC3479650

[B33] Adalimumab in FDA orphan drug product designation database http://www.accessdata.fda.gov/scripts/opdlisting/oopd/OOPD_Results_2.cfm?Index_Number=230306

[B34] Demir E Cary MP Paley S Fukuda K Lemer C Vastrik I Wu G D'Eustachio P Schaefer C Luciano J The BioPAX community standard for pathway data sharing Nat Biotechnol 2010 14 9 935 942 10.1038/nbt.1666 20829833 PMC3001121

[B35] Babur O Demir E Gonen M Sander C Dogrusoz U Discovering modulators of gene expression Nucleic Acids Res 2010 14 17 5648 5656 10.1093/nar/gkq287 20466809 PMC2943625

